# 

**Published:** 1888-09-01

**Authors:** 


### CONTENTS.

**A New Inquisition** - 349

**The Editor's Letter-box** - 350

**The Claims of Hospitals.** By R. BRUDENELL CARTER, F.R.C.S. - 351

**Workmen's Payments to Sunderland Hospitals.** By A. W. WEBSTER, Chairman of Workmen's Governors Sunderland Infirmary - 352

**The Doctor's Pulpit.**—Marriage—or What? - 353

**The Immediate Treatment of Accidents and Sudden Illness.** By Sir WILLIAM MOORE, K.C.I.E. - 354

**Turning Over New Leaves.**—"In Hot Haste," etc. - 355

**Notes and News** - 356

**Everybody's Page** - 358

**Annotations.**—Ambulances Still Wanted.—The Money Value of a Live Man.—Poor John Aldcroft! - 359

**Hints for the Sick-room.** By M. M BASIL, M.A., M.B. 360

**An Ishmaelite.** By LESLIE KEITH, Author of "The Chilcotes," "East and West," etc. - 361

**The Student's Column** - 363

**Amusements with Profit for all Ages** - 364

### EXTRA SUPPLEMENT.—THE NURSING MIRROR.

En Passant.—Where Women Kill Themselves.—The Pension Fund.—Remember the Nurses —All Saints' Sisterhood.—The Strange Drug "Friction."—The Zenana Medical College.—Queen Victoria Nurses' Institute - lxxxv
Original Articles.—Peculiar Patients.—The Crimea - lxxxvi
Letters from a Probationer.—I. - lxxxvii
Appointments. - lxxxvii
One Day at a Time - lxxxvii
Everybody's Opinion - lxxxviii
Notes and Queries —Vacancies.—Exchange and Mart - lxxxviii

